# Metabolic regulation of regulatory T cells: mechanisms, heterogeneity, and implications in disease

**DOI:** 10.3389/fimmu.2025.1729690

**Published:** 2025-12-12

**Authors:** Feven Getachew, Junhui Hu, Mehdi Benamar

**Affiliations:** 1 Division of Immunology, Boston Children’s Hospital, Boston, MA, United States; 2 Department of Pediatrics, Harvard Medical School, Boston, MA, United States; 3 Department of Otolaryngology Head and Neck Surgery, Shanghai Key Laboratory of Sleep Disordered Breathing, Otolaryngological Institute of Shanghai Jiao Tong University, Shanghai Sixth People’s Hospital Affiliated to Shanghai Jiao Tong University School of Medicine, Shanghai, China; 4 Institute of Regenerative Medicine and Biotherapy, INSERM, University of Montpellier, Montpellier, France

**Keywords:** regulatory T cells, Foxp3, metabolism, immune tolerance, diseases

## Abstract

Regulatory T (Treg) cells are essential for maintaining immune tolerance, preventing autoimmune responses, and supporting tissue repair. Tregs employ a flexible and diverse metabolic program that includes glycolysis, oxidative phosphorylation (OXPHOS), fatty acid oxidation, and lipid metabolism compared to conventional T cells, which largely rely on glycolysis to fuel their proliferation and function. This flexibility allows Tregs to adapt in different tissue environments while sustaining their suppressive activity. Thymic-derived (tTregs), peripheral (pTregs), and induced (iTregs) exhibit distinct metabolic profiles that influence their stability, proliferation, and suppressive capacity. These metabolic pathways are controlled by key regulators such as mTOR, LKB1, and Foxp3, while environmental cues, including nutrient availability, hypoxia, and microbiota-derived metabolites, further shape Treg function. Dysregulation of these pathways can compromise tolerance and contribute to immune-mediated diseases, chronic infections, cancer, and metabolic disorders. In this mini review, we summarize recent insights into the heterogeneity of Treg metabolism, highlighting how metabolic reprogramming underpins their immunoregulatory roles. We also explore therapeutic opportunities for targeting Treg metabolism and discuss future directions leveraging single-cell and spatial technologies to map context-specific metabolic programs *in vivo*.

## Introduction

Each organ system in the human body presents specific functions, and the immune system is no exception. One of its primary roles is to protect against invasion and destruction by pathogens distinguished as “non-self” antigens, while simultaneously tolerating host-derived “self” components (1–3). A breakdown of this delicate balance can result in the loss of immune tolerance and the initiation of aberrant immune responses against self-tissues, which are characteristic of autoimmune diseases (2, 4). Treg cells, a specialized subset of CD4^+^ T cells, play an essential role in controlling this balance by suppressing excessive immune responses and promoting immune homeostasis (3). Tregs represent approximately 5–15% of peripheral CD4^+^ T cells and are characterized by expression of CD25, the transcription factor Foxp3, and low or absent CD127 in humans (3, 5). Importantly, Tregs can exert suppressive effects by several immunosuppressive mechanisms, including the secretion of immunoregulatory cytokines, cytotoxicity, metabolic modulation, and modulation of antigen-presenting cells’ function (6, 7). Beyond immunosuppression, Tregs also contribute to tissue repair (8–13). The discovery of Tregs and their reliance on Foxp3 was initially highlighted by studies of the scurfy mouse and immune dysregulation, polyendocrinopathy, enteropathy, X-linked (IPEX) syndrome in humans, both caused by mutations in the Foxp3 gene (5, 14–16). These observations established Foxp3^+^ Tregs as indispensable guardians of self-tolerance. More recently, it has become clear that their function depends not only on transcriptional regulation but also on specialized metabolic programs (17, 18). Conventional T cells predominantly favor glycolysis and glutaminolysis to support rapid proliferation and effector functions (19, 20). In contrast, Tregs exhibit a more versatile metabolic program, dynamically shifting between glycolysis, oxidative phosphorylation (OXPHOS), lipid metabolism, and amino acid utilization depending on their differentiation state and microenvironmental cues (17, 19). This metabolic plasticity underpins their ability to exert diverse suppressive functions. In this mini review, we discuss the molecular underpinnings of Treg metabolism, highlight the heterogeneity between Treg subsets, and consider the implications of metabolic regulation in health and disease.

## Heterogeneity of Treg metabolism

### Thymic versus peripheral Tregs

**Figure 1:**
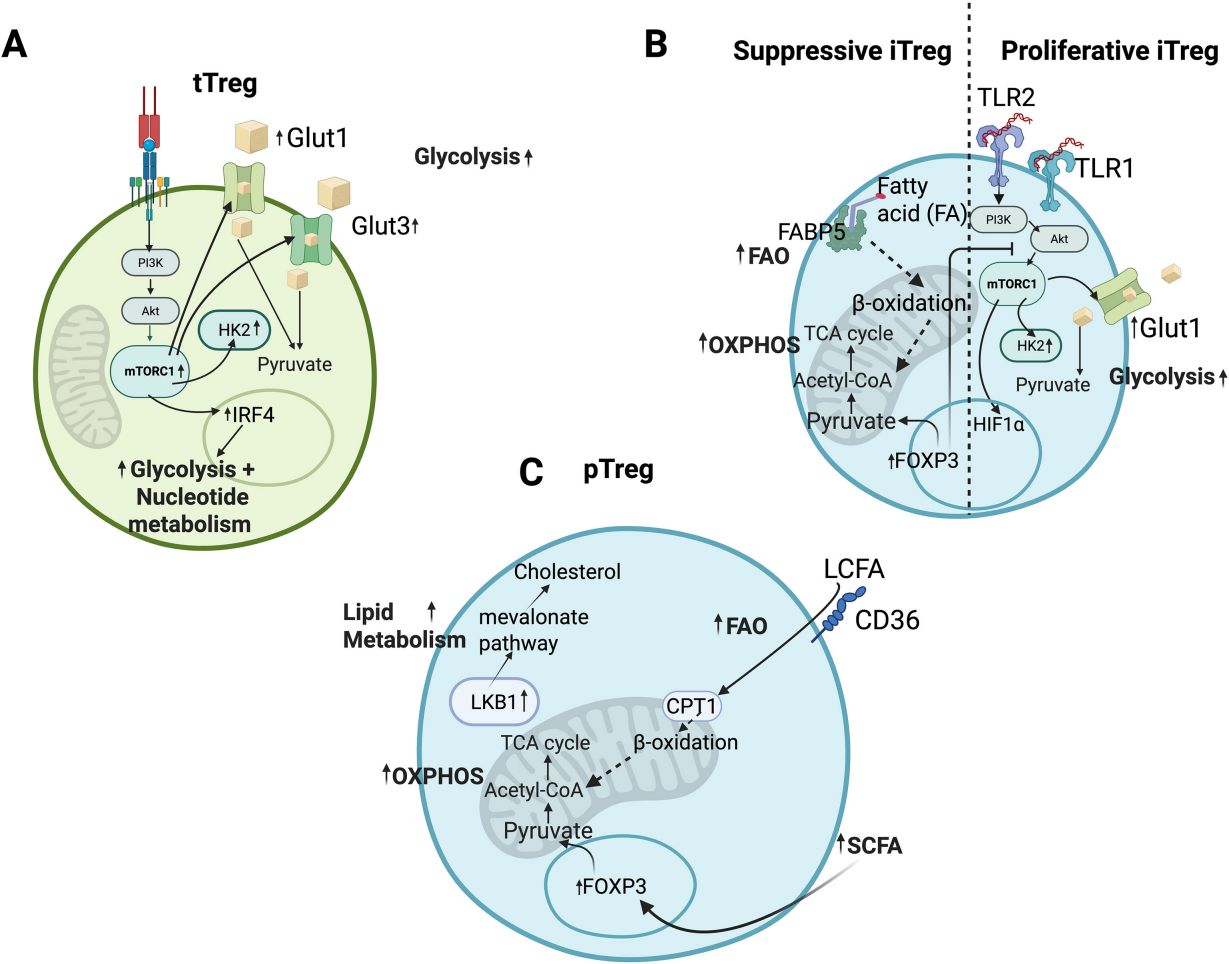
Heterogeneity of Treg metabolism. **(A)** Thymic Treg (tTreg) cells engage glycolysis through upregulation of Glut1 and Glut3, enhancing glucose uptake and activating PI3K–Akt–mTORC1 signaling. This pathway induces expression of hexokinase 2 (HK2), a rate-limiting enzyme in glycolysis. In peripheral tissues, antigen-driven activation of tTreg cells promotes mTOR–IRF4 signaling, further enhancing glycolysis and nucleotide metabolism. **(B)** Suppressive induced Treg (iTreg) cells (left) exhibit elevated Foxp3 expression, which reprograms cellular metabolism toward oxidative phosphorylation (OXPHOS). Upregulation of fatty acid–binding protein 5 (FABP5) facilitates mitochondrial fatty acid transport, promoting fatty acid oxidation (FAO) and increasing acetyl-CoA availability for OXPHOS. In contrast, proliferative iTreg cells (right) display TLR-dependent PI3K–Akt–mTORC1 activation, leading to increased Glut1 and HK2 expression, enhanced glycolytic flux, and induction of HIF-1α, which drives transcription of glycolytic genes. **(C)** Peripheral Treg (pTreg) cells (left) undergo Foxp3-dependent metabolic reprogramming toward OXPHOS, accompanied by activation of the mevalonate pathway via upregulation of liver kinase B1 (LKB1). Additionally, pTreg cells exhibit enhanced FAO through CD36-mediated uptake of long-chain fatty acids.

Thymic Tregs (tTregs) originate during T cell ontogeny in the thymus and are selected for high-affinity recognition of self-MHC molecules without crossing the threshold for deletion. Peripheral Tregs (pTregs) arise from conventional CD4^+^ T cells *in vivo* under tolerogenic conditions, while induced Tregs (iTregs) are generated *in vitro* from naive CD4^+^ T cells cultured in the presence of IL-2 and TGF-β (21, 22). Interestingly, tTregs and iTregs display distinct metabolic signatures (**Figures 1A, B**). Indeed, mouse tTregs cultured in IL-2 for three days exhibit high glycolytic activity, whereas iTregs induced with IL-2 and TGF-β display reduced glycolysis. Enhanced glycolysis in tTregs is associated with increased expression of glycolytic regulators such as HK2, Glut1, and Glut3. Addition of TGF-β to tTregs suppresses glycolysis, as indicated by reduced extracellular acidification rate (ECAR), decreased expression of glycolytic enzymes, and inhibition of the PI3K/Akt/mTOR pathway, which collectively impair their proliferation and suppressive capacity (23). In contrast, CD4-specific Glut1 deletion reduces conventional Foxp3^−^ CD4^+^ T cells but has little effect on Foxp3^+^ Tregs, suggesting that tTregs can rely on alternative metabolic pathways (24). These findings highlight the complexity of Treg metabolic requirements.

### Glycolysis in Treg proliferation and function

Glycolysis provides rapid ATP and biosynthetic intermediates necessary for cell growth. Previous studies have emphasized the importance of mTOR activation as an evolutionarily conserved requirement for T cell development and differentiation (25, 26). TLR1 and TLR2 signaling induce the PI3K–Akt–mTORC1 axis, which enhances mouse iTreg cells’ proliferation and glycolytic activity, as measured by increased ECAR and Glut1 expression (**Figure 1B**). However, these highly glycolytic iTregs show reduced suppressive capacity (27). Foxp3 itself appears to antagonize glycolysis (17). Ectopic expression of Foxp3 in naive T cells reduces glucose uptake, GLUT1 and HK2 expression, and Akt/mTOR phosphorylation, while increasing oxygen consumption and pyruvate oxidation. This metabolic shift diverts cells toward OXPHOS, supporting suppressive function rather than effector activity (27). Moreover, HIF-1α, a transcription factor stabilized under hypoxic and glycolytic conditions, favors Th17 differentiation while inhibiting Treg generation, illustrating the reciprocal balance between metabolic and immunological fates (28). While mTOR signaling is dispensable for iTreg differentiation *in vitro* (29), *in vivo* studies reveal its necessity for maintaining pTregs. It also promotes activation of peripheral tTreg to effector Treg status through regulation of IRF4, particularly in mucosal tissues which further promote glycolysis and nucleotide metabolism (30)(**Figure 1A**). In human Tregs, glycolysis is also critical. Freshly isolated, unmanipulated human Tregs demonstrate a higher dependence on glycolysis and express higher levels of mTOR compared to Tconv cells. Inhibition of mTOR using 2-DG impairs the suppressive function of Treg and reduces Foxp3 expression level in human Treg (31). Deletion of mTORC1 in Tregs leads to severe inflammatory disease, reduced mitochondrial fitness, and impaired metabolic reprogramming (32). These findings underscore that glycolysis can support Treg proliferation but may compromise suppressive function unless carefully balanced by other pathways.

### OXPHOS and lipid metabolism in Treg homeostasis

Beyond glycolysis, Tregs rely heavily on mitochondrial metabolism (**Figure 1C**). Deficiency in respiratory chain complex III within Treg cells leads to the accumulation of succinate and 2-hydroxyglutarate (2-HG) (33). These metabolites competitively inhibit TET enzymes by displacing their cofactor, α-ketoglutarate, causing hypermethylation and subsequent silencing of key immunosuppressive genes like PD-1, CD73, and TIGIT (34, 35). Ultimately, this epigenetic silencing impairs the cell’s ability to produce critical co-inhibitory molecules and ectoenzymes, severely decreasing the Treg’s immunosuppressive function. Foxp3 itself directly links transcriptional programming to mitochondrial metabolism. By promoting OXPHOS and FAO, Foxp3 enhances spare respiratory capacity and prevents fatty acid-induced apoptosis. Inhibition of fatty acid binding protein 5 (FABP5) disrupts mitochondrial integrity, reduces OXPHOS, and skews Treg metabolism toward glycolysis, compromising their suppressive function (36).

Liver kinase B1 (LKB1), also known as STK11, is a key metabolic sensor in Tregs. LKB1 deficiency leads to reduced mitochondrial oxygen consumption, ATP production, and mitochondrial mass, resulting in impaired Treg survival and function both *in vitro* and *in vivo* (37, 38). Metabolomic profiling shows that TCA cycle intermediates and fatty acid oxidation (FAO) products are markedly reduced upon LKB1 deletion (39). LKB1 also regulates cholesterol biosynthesis via the mevalonate pathway. Tregs lacking LKB1 or the key enzyme HMGCR show impaired lipid metabolism, increased inflammatory cytokine production, and systemic autoimmunity, which can be partially rescued by mevalonate supplementation (38). These results emphasize that lipid metabolism is not only a source of bioenergetics but also provides critical intermediates for maintaining immune tolerance. CD36, a fatty acid transporter, has been identified as another important metabolic adaptation in intratumoral Tregs. By facilitating uptake of long-chain fatty acids and oxidized lipoproteins, CD36 enhances PPAR-β signaling, mitochondrial fitness, and NAD^+^/NADH balance, thereby promoting Tregs survival in nutrient-poor tumor microenvironments (40). Together, these findings demonstrate that Tregs require intact mitochondrial and lipid metabolic pathways for long-term survival, stability, and function.

### Metabolic crosstalk with the microenvironment

Treg metabolism is highly sensitive to external cues. Nutrient availability, oxygen tension, and microbial metabolites all shape Tregs function. Short-chain fatty acids (SCFAs), such as acetate, propionate, and butyrate produced by gut microbiota, promote Tregs differentiation via epigenetic regulation of Foxp3 and enhanced FAO (26). Propionate facilitates Treg differentiation *in vitro* via the GPR43-HDAC6 axis, which correlates with an elevated Treg/Th17 ratio *in vivo* in mice (41). Similarly, butyrate and its derivatives exert potent pro-regulatory effects. Treatment with methyl butyrate fosters Treg development and IL-10 secretion in the draining lymph nodes of EAE mice and also directly promotes Treg differentiation *in vitro* (42). Furthermore, evidence indicates that butyrate itself helps regulate the Th17/Treg balance by activating PPARγ, a process which subsequently promotes the upregulation of CD36 and Foxp3 *in vitro* (43) (**Figure 1C**). Tryptophan metabolites, generated by commensals through the indoleamine-2,3-dioxygenase (IDO) pathway, also favor Treg stability while suppressing effector T cell responses. These findings highlight that Treg metabolism is not cell-autonomous but is deeply influenced by interactions with the microbiome and tissue-specific niches. Such insights raise the possibility that microbiome-targeted therapies could modulate Treg metabolism to treat inflammatory diseases.

## Treg metabolism in disease

### Autoimmunity and chronic inflammation

**Figure 2:**
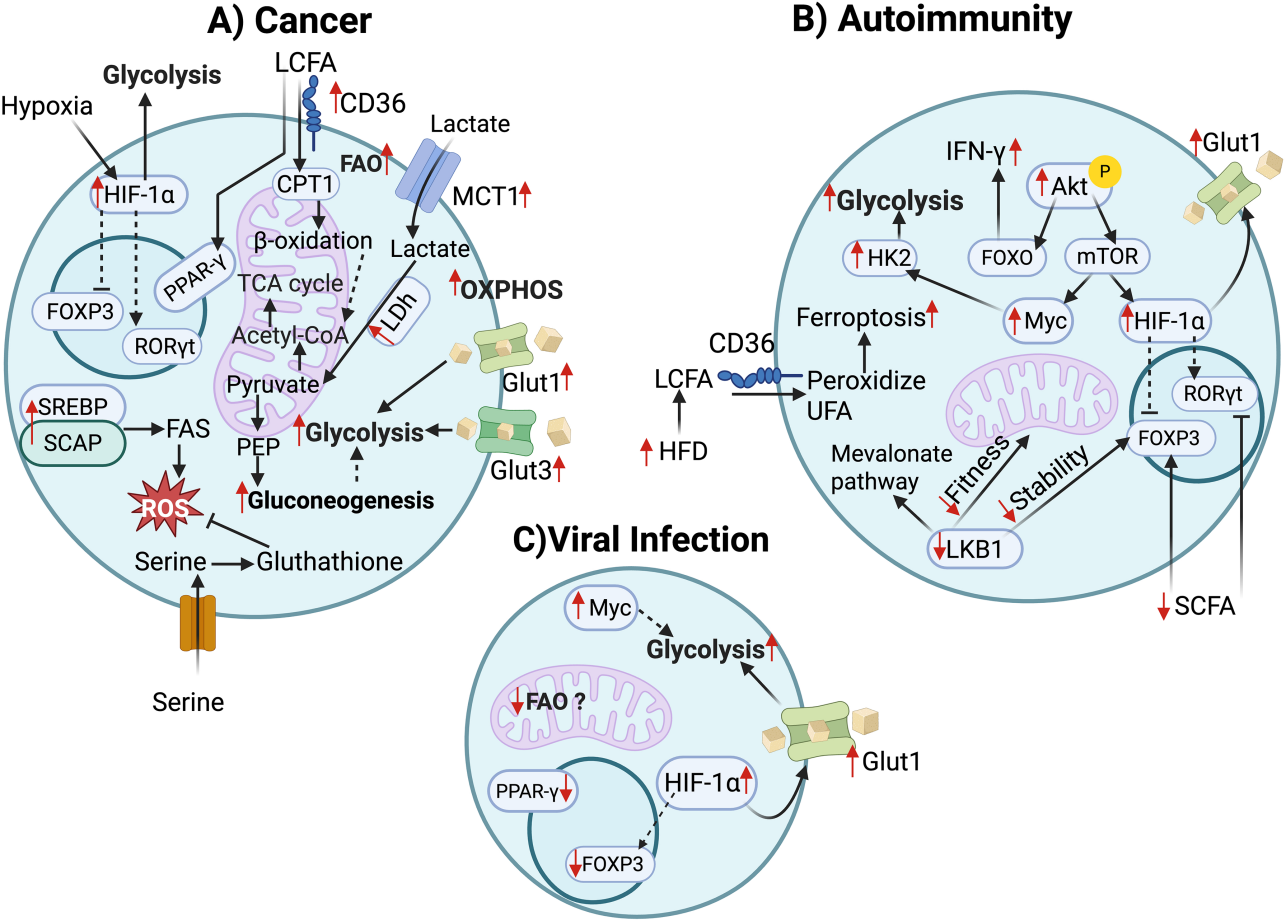
Treg metabolic changes in the context of disease. **(A)** Tumor infiltrating Tregs (TI-Tregs) have several adaptations: TI Tregs upregulate MCT1 for lactate uptake and Lactate dehydrogenase (LDh) for conversion of Lactate to pyruvate. Additionally, lactate derived PEP fuels TI-Treg proliferation. TI-Tregs upregulate Glut1 and Gult3 for glucose uptake and entry to OXPHOS. Hypoxic conditions induce HIF-1α upregulation and potential destabilization of Foxp3 causing TH17 skewing limiting suppressive function although this requires further studies. CD26 mediated LCFA uptake increases fatty acid oxidation and PPARγ signaling further supporting mitochondrial fitness and OXPHOS. TI-Tregs upregulate SCAP mediated SREBP activation to induce Fatty acid synthesis. Tregs achieve reduction of ROS associated with lipid metabolism by serine dependent glutathione (GSH) synthesis. **(B)** In high-fat diet induced colitis, Tregs uptake long chain fatty acids through CD36 leading to increased peroxide unsaturated fatty acid induced ferroptosis. In Multiple sclerosis, autoimmune hepatitis and type-1 diabetes, increased Akt phosphorylation and mTOR signaling induce expression of HIF-1α and Myc which results GLUT1 receptor and hexokinase 2 upregulation to facilitate glycolytic reprograming. Akt phosphorylation also induces FOXO further exacerbating IFNγ production. Reduced SCFA exacerbates Th17/Treg skewing in diseases such as IBD. Reduced LKB1 promotes reduced metabolic fitness and OXPHOS related gene expression as well as Foxp3 stability leading to autoimmune disorder such as acute graft-versus-host disease (aGVHD). **(C)** In Viral infection, Tregs upregulate Glut 1, and glycolytic genes HIF-1α and Myc while decreasing genes associated with. PPARγ signaling and FAO although additional studies are required to in this realm.

Metabolic dysregulation of Tregs contributes to autoimmune pathology (**Figures 2A–C**). For example, constitutive Glut1 activation in Tregs reduces Foxp3 expression and impairs suppression in colitis models (27), whereas Glut1 deletion has little effect on Treg function (24). Obesity is associated with increased mTOR activation, glycolytic gene expression, and reduced Treg frequency in adipose tissue, contributing to insulin resistance and chronic inflammation (44). In multiple sclerosis (MS) and experimental autoimmune encephalomyelitis (EAE), Tregs display impaired mitochondrial metabolism, reduced spare respiratory capacity, and altered lipid handling, correlating with disease severity. Importantly, evidence from human studies highlights a distinct role for glycolysis in maintaining Treg stability. De Rosa et al. demonstrated that glycolysis controls the induction of human Tregs by modulating FOXP3 exon 2 splicing variants (45). Impaired glycolysis and reduced Foxp3 exon 2 (Foxp3-E2) expression were also observed in iTregs from patients with relapsing-remitting multiple sclerosis (RRMS) and type 1 diabetes (T1D) (45). Studies have shown that an increase of metabolic sensors such as HIF-1α is responsible for Th17/Treg imbalance that causes disease pathogenesis in EAE (46). Finally, mice models with Treg-specific deletion of LKB1 further demonstrating the relationship between metabolic dysregulation in Treg, Treg stability, and function in inducing immune imbalance (47). These observations underscore the potential of targeting Treg metabolism to restore tolerance in autoimmunity.

### Cancer

In the tumor microenvironment (TME), Tregs adapt their metabolism to survive under hypoxic and nutrient-poor conditions (**Figures 2A, B**). Unlike effector T cells, which suffer from nutrient competition and exhaustion, mouse Tumor-infiltrating Tregs (TI-Tregs) upregulate lactate transporters (MCT1) and lactate dehydrogenase (LDh) to metabolize extracellular lactate into pyruvate for OXPHOS (48). Additionally, TI-Tregs also utilize Lactate to generate Phosphoenolpyruvate (PEP), a precursor of pyruvate suggesting increased gluconeogenesis to fuel their proliferation (48). Interestingly, mouse TI-Tregs upregulate HIF-1α due to hypoxic conditions, which enhances their glycolytic-driven migration but compromises their suppressive function (49, 50). To maintain suppressive function, mouse TI-Tregs utilize fatty acid and lipid metabolism; indeed, TI-Tregs upregulate CD36 expression, which enhances fatty acid oxidation and the PPAR signaling pathway responsible for mitochondrial fitness and adaptation to lipid metabolism (40). In humans, TI-Tregs also displayed a gene signature oriented to glycolysis and lipid biosynthesis (51). Additionally, mouse studies also show that TI-Tregs upregulate SCAP/SREBP signaling, leading to *de novo* lipid biosynthesis, including fatty acids and cholesterols (52, 53). To withstand the inevitable ROS build-up associated with increased lipid metabolism, TI-Tregs upregulate antioxidant molecule synthesis such as serine-dependent glutathione (GSH) synthesis (50, 54). This mechanism is conserved in humans, as pharmacological inhibition of GSH synthesis in human iTregs led to loss of suppression and reduced FoxP3 expression (54). Furthermore, TI-Tregs compete with effector T cell to uptake the limited glucose in the TME by upregulating GLUT1 and GLUT3 receptors (50). These adaptations allow them to maintain suppressive capacity and dampen anti-tumor immunity, representing a major barrier to immunotherapy. Lipid metabolism also plays a role in TI-Tregs; TI-Tregs display a metabolic preference for lipid utilization compared to their circulating counterparts in different types of human cancer. Functional studies also confirm that TI-Tregs uptake more fatty acids in mouse cancer models (40). Targeting Treg metabolism, for example, through inhibition of CD36 or MCT1, is therefore being explored as a strategy to selectively impair tumor-infiltrating Tregs while sparing systemic tolerance.

### Infectious disease

In chronic viral infections such as HIV and HCV, Tregs expand and display altered metabolic signatures, contributing to viral persistence by suppressing effector T cell responses (**Figure 2C**). In chronic HIV patients undergoing AntiRetroviral Therapy (ART), a study revealed that gut Treg cells from patients had upregulated glycolysis-related genes while downregulating genes associated with OXPHOS and FAO, leading to Treg cells expansion with decreased function (55). A study on Tregs of Long-term non-progressor (LTNPs), a small group of HIV-infected individuals with decreased viral load without ART, has increased glycolytic genes such as Myc and HIF-1α and glycolytic receptors such as Glut1, while having decreased FAO and PPAR signaling, which suggests increased expansion but decreased Treg suppressive capacity (56). Conversely, in acute infection, Tregs may prevent immunopathology by limiting excessive inflammation (57). Yet, there is a large gap in knowledge regarding establishing a direct relationship between alteration of Treg metabolism under chronic and acute infections. Such investigations can provide bases for strategic manipulation of Treg metabolism to balance pathogen clearance with tissue protection.

### Future directions and therapeutic implications

The field of Treg metabolism is rapidly evolving, with several promising directions. Therapeutic modulation of key metabolic pathways, such as mTOR, AMPK, and FAO, offers the potential to enhance or suppress Treg function depending on the disease context; for instance, metformin, an AMPK activator, promotes FAO and supports Treg survival in autoimmune settings. However, systemic manipulation of metabolism poses risks due to widespread effects, highlighting the need for precision targeting strategies that are tissue-specific or context-dependent. Ultimately, these insights hold strong potential for clinical translation, informing the design of adoptive Treg therapies for autoimmunity and transplantation, as well as strategies for selectively targeting Tregs to improve cancer immunotherapy.

## Conclusion

Regulatory T cells are indispensable for immune tolerance and tissue homeostasis. Their ability to adapt metabolism between glycolysis, OXPHOS, and lipid pathways underlies their functional heterogeneity across contexts such as the thymus, peripheral tissues, and tumors. Dysregulated metabolism compromises their suppressive function and contributes to pathology in autoimmunity, cancer, chronic infection, and obesity. A deeper mechanistic understanding of Treg metabolism, combined with advances in technology, is likely to open new avenues for therapeutic intervention. Targeting metabolic pathways offers both opportunities and challenges but holds great promise for fine-tuning immune responses in diverse disease settings.
